# The Ins and Outs of Viral Infection: Keystone Meeting Review

**DOI:** 10.3390/v6093652

**Published:** 2014-09-25

**Authors:** Sara W. Bird, Karla Kirkegaard, Mavis Agbandje-McKenna, Eric O. Freed

**Affiliations:** 1 Department of Microbiology and Immunology, Stanford University School of Medicine, Stanford, CA 94305, USA; E-Mails: birdie@stanford.edu (S.W.B.); karlak@stanford.edu (K.K.); 2 Department of Biochemistry and Molecular Biology, University of Florida, Gainesville, FL 32610, USA; E-Mail: mckenna@ufl.edu; 3 Virus-Cell Interaction Section, HIV Drug Resistance Program, Center for Cancer Research, National Cancer Institute, Frederick, MD 21702, USA

**Keywords:** Keystone Symposium, virus infection, Ebola, HIV-1, VZV, HSV, HCV

## Abstract

Newly observed mechanisms for viral entry, assembly, and exit are challenging our current understanding of the replication cycle of different viruses. To address and better understand these mechanisms, a Keystone Symposium was organized in the snowy mountains of Colorado (“The Ins and Outs of Viral Infection: Entry, Assembly, Exit, and Spread”; 30 March–4 April 2014, Beaver Run Resort, Breckenridge, Colorado, organized by Karla Kirkegaard, Mavis Agbandje-McKenna, and Eric O. Freed). The meeting served to bring together cell biologists, structural biologists, geneticists, and scientists expert in viral pathogenesis to discuss emerging mechanisms of viral ins and outs. The conference was organized around different phases of the viral replication cycle, including cell entry, viral assembly and post-assembly maturation, virus structure, cell exit, and virus spread. This review aims to highlight important topics and themes that emerged during the conference.

## 1. Keynote

One of the highlights of the meeting was the keynote address given by **Geoffrey Smith** (University of Cambridge, UK). He detailed the history of smallpox infection and pathogenesis and highlighted the key features that made the eradication efforts so successful while addressing what may hinder us in the present and future from successful eradication campaigns targeting other deadly viral pathogens. As a continuation of previous work from Smith’s lab [1], he also detailed an exciting mechanism for repulsion of cell entry of vaccinia virus in cells that are already infected; this mechanism facilitates more rapid cell-to-cell spread of the virus.

## 2. Mechanisms of Cell Entry

**Sean Whelan** (Harvard Medical School) opened the session on viral entry by discussing his work with vesicular stomatitis virus (VSV), which he uses as a model to study the replication of pathogenic negative-strand RNA viruses. This is accomplished by expressing glycoproteins from the virus of interest, in this case rabies virus, on the surface of VSV to create a recombinant VSV (rVSV). With this system, Whelan demonstrated that, on binding to its receptor, rVSV is internalized through altered vesicles resembling clathrin-coated pits [2]. Actin is required for the internalization process as inhibitors of actin were shown to abrogate entry of the virus. Whelan also used VSV pseudotypes bearing Ebola virus glycoprotein to screen for host factors required for Ebola entry. This approach identified the lysosomal cholesterol transporter, Niemann-Pick C1 (NPC1), as an important co-factor in Ebolavirus entry [3,4].

Hepatitis C virus (HCV) entry requires the tight junction proteins claudin and occludin [5,6]. This would suggest a role for tight junctions as the entry site for HCV. However, earlier imaging studies of HCV entry from Glenn Randall’s lab using non-polarized cells failed to observe HCV entry at intra-cellular junctions [7]. **Yasmine Andonyadis** from the Randall lab (University of Chicago) took advantage of a polarized three-dimensional cell culture system with well-defined tight junctions. Using single-particle tracking of HCV and 3D polarized cells, it was shown that HCV traffics to the tight junctions in a process dependent on actin, co-localizes with claudin and occludin, and enters cells by clathrin-mediated endocytosis. Epidermal growth factor receptor (EGFR) signaling was required for clathrin-mediated endocytosis of HCV, but was dispensable for HCV trafficking to the tight junction. These data highlight the importance of utilizing relevant cell types to study processes of the viral life cycle.

**Matthew Evans** (Icahn School of Medicine, Mount Sinai) also presented his work on HCV entry in polarized cell systems. Induced pluripotent hepatic stem cells from pigtail macaques are capable of supporting the entire replication cycle of HCV; however, infection inefficiencies were identified at the point of viral entry, specifically due to an ineffective form of CD81. This was overcome by infecting cells with a strain of HCV with less stringent requirements for CD81 or by ectopically expressing human CD81. This work suggests that genetically modified pigtail macaques could potentially provide a relevant *in vivo* model to study HCV infection, pathogenesis, and treatment [8].

It is widely believed that alphaviruses enter cells by receptor-mediated endocytosis followed by a membrane fusion event in the endosomal compartment. Using electron microscopy, **Dennis Brown** (North Carolina State University) showed that alphavirus entry goes against the dogma of receptor-mediated endocytosis. Instead, viral capsids, devoid of their RNA genomes, were found on the cell surface following antibody labeling along with a pore structure where the virion and plasma membrane meet. These data suggest a new model for alphavirus entry, whereby viral attachment leads to conformational changes allowing for RNA entry by direct penetration through a pore structure formed by the virus itself [9].

One of the major pitfalls in studying the viral replication cycle in 2D culture systems is the inability to properly recapitulate the tissue structure and architecture viruses are exposed to *in vivo*. **Carolyn Coyne** (University of Pittsburgh) presented a fascinating new model for studying viral entry and infection that employs a 3D cell culture system. In this system, applied here to study the picornavirus coxsackie B3, intestinal cells are grown on collagen-coated beads and cultured in a rotating wall vessel bioreactor; these cells display many of the hallmarks of intestinal epithelial cells, including polarity and tight junctions. This model system will have important consequences for understanding how viruses infect tissues versus how they behave in standard tissue culture, as the 3D cell models seem to be differentially permissive to viral infection compared to cells grown in 2D.

As cell entry is a critical step in the replication cycle of all viruses, it is an attractive target for antivirals. To investigate the viral and host factors required for poxvirus infection, **Jason Mercer** (ETH Zurich) developed parallel host cell- and virus-targeting high-throughput RNAi screens using vaccinia virus. Combined, the screens identified a novel mechanism for genome uncoating involving the ubiquitin-proteasome system [10], as well as a viral uncoating factor, D5, a AAA + ATPase [11]. These results highlight the utility of large-scale screens for identifying new viral targets.

## 3. Virion Assembly and Post-Assembly Maturation

**Andrea Gamarnik** (Fundacion Instituto Leloir, Argentina) began this session by highlighting her work with Dengue virus (DENV), discussing the capsid determinants for RNA encapsidation. The DENV capsid is known to accumulate in lipid droplets with the help of cellular factors including the coat protein complex COPI and ADP ribosylation factor (ARF), but it is unclear why. To investigate the determinants of the DENV capsid that are important for both encapsidation and uncoating, selective 2’-hydroxyl acylation analyzed by primer extension (SHAPE) analysis was performed to gather detailed structural information about the capsid RNA. With this information, reporter DENVs were designed with specific mutations in the capsid without affecting cis-acting RNA elements for genome replication. The analysis revealed two clusters of basic residues at the N-terminus of the capsid that were completely indispensable for viral infectivity. In addition, reminiscent of some aspects of Jason Mercer’s work with vaccinia virus (see above), Gamarnik showed that DENV uncoating depended upon host cell ubiquitination machinery; however, in this case, proteasome-dependent degradation of capsid was not required to release the viral RNA for subsequent translation.

It is well established that HCV particles interact with serum lipoproteins [12,13] but how and why this interaction takes place remains unclear. It has even been proposed that HCV is a lipo-viro-particle. **Brett Lindenbach** (Yale) presented genetic and biochemical evidence that apolipoprotein E (ApoE), found in very low-density lipoproteins, is necessary and sufficient for HCV assembly. Mutations in ApoE, as well as use of ApoE-deficient Huh7 cells, abrogated HCV infectivity. Furthermore, the structure of ApoE resembles a tether, suggesting that ApoE links HCV particles to lipoproteins. In support of this model, biochemical evidence was presented indicating that ApoE does indeed act as a tether.

During the maturation process of non-enveloped virus particles, many conformational changes in the capsid occur, often producing a membrane-active lytic peptide via an autocatalytic cleavage event. To study virus particle maturation, **Jack Johnson** (Scripps Research Institute) and his group use the simple non-enveloped Nudaurelia capensis ω (NωV) virus as a model. NωV provides an attractive model to study the kinetics of capsid cleavage and particle maturation because the virus can be purified and manipulated *in vitro* by altering the pH and the icosahedral capsid is composed of four identical gene products with drastically different cleavage rates. It was shown that the cleaved peptides for each of the four capsid proteins serve different functions. The peptides associated with the C and D subunits are involved in particle stabilization while those associated with the A subunits possess the lytic activity. Using NωV as a model reveals how tightly coordinated viral particle maturation is and that identical peptide sequences can perform vastly different functions [14].

Many viruses undergo a maturation step after particle release from the infected cell. In the case of HIV-1, particle maturation involves the sequential, step-wise cleavage of the major viral structural protein, Gag, by the viral protease. Protease-mediated Gag cleavage leads to a morphological rearrangement of the virion from the non-infectious, immature virion to a mature, infectious particle [15]. **Eric Freed** (National Cancer Institute, NIH) reported on recent progress in developing compounds that target a specific step in HIV-1 Gag processing, thereby blocking virion maturation. Initial studies in the Freed lab focused on the first-in-class maturation inhibitor dimethyl-succinyl betulinic acid (known as bevirimat) [16]. Work on a structurally distinct maturation inhibitor, PF-46396, provided important insights into the structure and function of the maturation inhibitor binding pocket [17]. Most recently, Freed and collaborators have focused on developing bevirimat analogs with greatly increased potency and breadth of activity against diverse strains of HIV-1.

## 4. Virus Structure

Studying the structure of viruses can reveal key details of the viral replication cycle.

**Erica Ollmann Saphire** (Scripps Research Institute) presented the idea of “viral protein origami” to challenge the idea that one protein yields one structure to elicit one function. To understand the multifunctionality of a particular protein, crystal structures of the Ebolavirus protein VP40 were obtained; further biochemical analysis revealed that VP40 can form three distinct structures with three distinct functions. VP40 can form a butterfly-shaped dimer, with the interface at the N-termini, which traffics to the cellular membrane. Once at the cellular membrane, electrostatic interactions lead to a rearrangement into a hexamer in which the VP40 proteins align in a parallel arrangement. Multiple hexamers form a filament that is essential for assembly and budding. Finally, a third VP40 structure forms when the hexamers assemble into zig-zag filaments. This structure is capable of binding viral RNA for regulation of transcription. Thus, VP40 is an example of a “transformer”, capable of adopting different structures with different functions at different times during infection [18].

**Mavis Agbandje-McKenna** (University of Florida) utilized X-ray crystallography combined with other biophysical approaches and molecular biology to identify distinct capsid features of the *Parvoviridae*, specifically Adeno-associated virus (AAV), required for viral entry. Capsid dynamics was observed to play a major role in the process. In addition, the studies uncovered novel capsid functions, such as protease activity and a role in genome transcription.

The bacteriophage BPP-1 infects *Bordetella* species of bacteria, causative agents of whooping cough, and has been targeted as an ideal candidate for phage display due to its diversity-generating retroelements (DGR). However, efforts to engineer the bacteriophage for phage display are hindered by the lack of a high-resolution structure. **Hong Zhou** (UCLA) presented the structure of the bacteriophage BPP-1 head at the remarkable resolution of 3.5 angstroms. Non-enveloped virus capsid proteins typically adopt one of three structures: the β-jellyroll, the HK97 fold, and the double-stranded DNA virus shell protein folding [19]. The BPP-1 capsid atomic structure revealed a jellyroll fold for the cement protein and an HK97-like fold for its major capsid protein. This high-resolution structure illustrates the feasibility of using BPP-1 as a phage display system and for DGR engineering [19].

Structural studies can reveal a great deal about the ancestral lineage of seemingly unrelated proteins. **Felix Rey** (Institut Pasteur) and his group, in collaboration with B. Podbilewicz from Technion, Israel, found structural homology between the EFF-1 fusion protein of *C. elegans* and viral class II fusion proteins even though the two proteins have no sequence similarity. The crystal structure of EEF-1 at a 2.6-angstrom resolution revealed that it has the same 3D fold as the viral class II fusion proteins, suggesting an ancient exchange of genetic information between cells and viruses. However, whether the fusion protein is viral in origin or if it “stole” the protein from its host is more difficult to determine. Further structural characterization of EEF-1 revealed that it behaves similarly to a SNARE protein by anchoring in opposing membranes and zipping them together [20].

HIV entry is mediated by cleaved, trimeric envelope glycoprotein spikes, which are an attractive target for generating broadly neutralizing antibodies (bNAbs) but have proven difficult to characterize structurally. **John Moore** (Weill Medical College of Cornell University) discussed an approach to generate bNAbs, which involves generating envelope trimers. These trimers closely resemble spikes formed by the native trimer, are highly stable, and have been characterized structurally to a resolution of 6 angstroms [21,22]. This advanced characterization of the trimer spikes will not only further our understanding of HIV entry but may also provide more effective approaches for designing a structure-based HIV vaccine.

## 5. Cell Exit

A common exit strategy for many viruses is to lyse the host cell, thereby releasing the progeny virions. Bacteriophage lysis is accomplished by small membrane proteins of the “lysis cassette” including holins, which form aggregates and poke holes in membranes, and endolysins, which degrade the bacterial peptidoglycan. Holins fall into two different classes based on the size of the membrane pore they form. Canonical holins form holes on the micron scale, allowing for endolysin proteins to exit the cytoplasm. In contrast, pinholins form holes 2 nm in diameter where signal anchor release (SAR) endolysins are secreted, but still membrane-tethered. **Ryland Young** (Texas A&M University) presented evidence that, in addition to holins and endolysins, a third class of proteins, the spanins, is required for efficient lysis of the bacterial outer membrane. In the absence of spanins, progeny bacteriophage are trapped within spherical cells bounded by the outer membrane, which failed to be degraded by the spanins [23]. The spanins function like SNARE proteins by fusing the inner and outer bacterial membranes, which in conjunction with holin and endolysin function, serve to effectively lyse the bacterial cells.

The advent of sensitive and high-resolution microscopy techniques has provided a powerful set of tools to ask pointed questions about steps in the viral replication cycle, particularly entry and exit. Much is currently known about the alphavirus entry pathway, but viral egress remains poorly understood. **Maria Guadalupe Martinez** (Albert Einstein College of Medicine) used fluorescently-labeled Sindbis viruses (SINV) along with confocal and total internal reflection fluorescence microscopy to visualize virus budding and egress. The E2 transmembrane protein of SINV congregates in patches along the plasma membrane, as well as in filopodia-like extensions that were of two distinct types: tubulin negative and tubulin positive, the latter of which could transfer virus particles to new target cells [24].

The autophagy pathway, a recycling program found in all cells, is subverted by a number of viruses. Autophagy is often induced in virus-infected cells, but whether it is due to the cell clearing the virus, or the virus utilizing the pathway for its replication cycle, is under active investigation. **Charles Grose** (University of Iowa) showed that Varicella-Zoster Virus (VZV)-infected cells undergo very high levels of autophagy. Viral titers and cytopathic effects were drastically reduced by inhibitors of autophagy. Furthermore, efficient processing of VZV glycoproteins required for viral spread was decreased when ATG5, a critical autophagy protein, was depleted. Conversely, treatment of cells with trehalose, an mTOR-independent inducer of autophagy, accelerated both the spread of the virus and synthesis of viral proteins [25]. This work provides another example of a virus that has subverted the autophagy pathway to its benefit.

Another group of conserved proteins often subverted by viruses is the endosomal sorting complex required for transport (ESCRT), involved in multivesicular body biogenesis and cellular abscission during cytokinesis. The ESCRT machinery plays an important role in the exit of many enveloped viruses including the herpes simplex virus 1 (HSV-1) [26]; however, the mechanism by which HSV-1 recruits ESCRT proteins has remained unclear. **Juan Martin-Serrano** (King’s College London School of Medicine) used genetic approaches and time-lapse microscopy of HSV-1 mCherry in GFP-expressing cells to monitor spread of HSV-1 in real time. Knock down or chemical inhibition of ESCRT machinery greatly diminished viral spread. In a yeast two-hybrid screen, the HSV-1 protein UL47 was found to interact with multiple ESCRT proteins and deletion of UL47 impaired HSV-1 spreading. Therefore, UL47 may play an important role in recruiting the ESCRT machinery to the sites of HSV-1 exit.

**Rebecca Ellis Dutch** (University of Kentucky) reported on the transmission of human metapneumovirus (HMPV), which causes severe respiratory infections in infants and the elderly. To better understand the mechanism of HMPV spread, viral proteins, and cellular morphology were monitored in infected human lung cells. Cell-to-cell spread of most paramyxoviruses is generally thought to occur only by syncytium formation. However, the data presented in this talk demonstrated the formation of extensive networks of cell-associated filamentous virus and of cellular extensions in infected cells. Formation of these structures required elements of the cellular cytoskeleton. In addition, the extensions were observed between cells and contained all tested viral proteins, suggesting a novel form of cell-to-cell spread for HMPV.

The way in which viruses exit an infected cell can have a profound impact on how they enter new target cells. As **Hyeryun Choe** (Scripps Research Institute, Florida) described, phosphatidylserine (PS) is exposed on the outside of many enveloped viruses. Phagocytic cells respond to PS, found normally on apoptotic bodies, via T-cell immunoglobulin and mucin-domain-containing (TIM) receptors, and engulf these bodies. Choe’s group has shown that infection of many enveloped viruses, such as filoviruses and flaviviruses, is enhanced by these PS receptors, which often function in conjunction with primary viral receptors [27]. To determine whether or not a viral receptor is necessary for PS-mediated entry, Choe and colleagues infected CHO cells expressing TIM-1 but deficient for expression of the Ebolavirus receptor and found that Ebolavirus entry did not occur. However, blocking PS receptors on macrophages also prevented entry of Ebolavirus virus-like particles. These results suggest that PS receptors could provide a broad therapeutic target against infection of a diverse family of enveloped viruses.

## 6. Virus Spread

Viral spread is complex and dynamic, making it amenable to mathematical modeling. **Alan Perelson** (Los Alamos National Laboratory) presented ideas for improving upon existing models by taking into account the spatial structure of the infected tissue, more relevant for studying *in vivo* infections. Using laser capture microdissection of liver biopsy material to analyze spatial relationships of HCV-infected cells revealed the formation of small clusters of up to 50 infected cells. Measuring the viral RNA levels revealed that RNA amounts decreased outwards from the founder cell [28]. Based on the amount of RNA in each infected cell within a cluster, a model was developed to determine how long the infection had progressed. A separate model was applied to study the cell-to-cell spread of the virus in the presence or absence of anti-viral drugs as measured in longitudinal serum samples. The utility of this model is that it allows for the study of viral factors, cellular factors, and antivirals, and can be applied to data from existing clinical trials to better understand the efficacy of the drugs in limiting virus replication and spread.

To study the spread of alphaherpesviruses *in vitro*, **Matthew Taylor** (Montana State University) grew neurons in chambered culture slides; in this system, the neuronal cell body attaches at one end and individual axons extend between grooves to the other end of the chamber. Using a three-color viral infection assay combined with live-cell imaging of the neuronal cultures allowed for unique analysis of the dynamics of viral spread. When three, differentially colored viruses were added to the neuronal cultures, sections of single-color infected cells could be visualized. This suggests that a limited number of genomes are expressed in the newly infected cell and that there may be a population bottleneck to viral diversity [29]. Complementary to this work, **Lynn Enquist** (Princeton) presented *in vivo* imaging studies of infection by fluorescently-labeled alphaherpesvirus particles in mice to better understand the mechanisms and dynamics of how these viruses infect the peripheral nervous system (PNS). The results confirmed much of the data on viral particle movement obtained *in vitro* with cultured neurons with respect to velocity, although movement in axons was significantly more processive after *in vivo* infection. Surprisingly, however, the expected retrograde motion of the herpesvirus particles toward PNS cell bodies early during infection was dominated by anteretrograde movement back to the site of infection where these progeny virus particles can reseed innervating axons and infect more PNS neurons [30]. This type of “round trip” infection may explain some of the characteristic peripheral neuropathies associated with alphaherpesvirus infections [31].

The spread of enteric viruses through the body involves passage through the digestive tract, where they encounter diverse gut microbiota. **Julie Pfeiffer** (UT Southwestern) showed that enteric viruses require these gut bacteria for efficient replication and pathogenesis [32]. Poliovirions are stabilized and attach to their receptor with higher affinity upon exposure to lipopolysaccharide (LPS). A poliovirus mutant with poor LPS binding showed efficient cell attachment and replication but reduced stability in the feces of infected mice. The stabilization of the virion by LPS is, thus, likely to play an important role in transmission of virus particles to a new host. This is an interesting example of a group of viruses evolving dependency on the microbes in the surrounding environment for spread through and to a new host.

Viral subversion of the autophagy pathway, as Charles Grose demonstrated for varicella zoster virus (see above), was first shown for poliovirus. The cytoplasmic replication organelles created during infection with poliovirus closely resemble canonical autophagosomes. **Karla Kirkegaard** (Stanford University) presented evidence that the double-membraned organelles used for poliovirus replication may also provide a mechanism for the virus to escape from the cell prior to lysis as a type of unconventional secretion. Like many examples of unconventional secretion, confirmation of non-lytic spread requires the demonstration that a small amount of cellular lysis in the cellular population gave rise to the release of cytosolic material. Using quantitative time-lapse microscopy, poliovirus was shown to spread between cells in the absence of lysis. siRNA-mediated knockdown of autophagy protein LC3 reduced non-lytic intercellular viral transfer while pharmacological stimulation of the autophagy pathway caused more rapid viral spread in tissue culture and greater pathogenicity in mice. Using a live-dead stain, it was shown that the virus can spread from cell to cell prior to lysis of the originally infected cell in a manner dependent on the autophagy pathway. It is likely that other non-enveloped viruses and cytoplasmic components can also use this pathway for non-lytic intercellular spread [33].

**Akira Ono** (University of Michigan) presented work from his group on the retroviral structural protein Gag and its role in HIV assembly and spread. The matrix (MA) domain of Gag directs HIV assembly to the plasma membrane via an interaction between its polybasic region and an acidic phospholipid PI(4,5)P_2_, but the mechanisms determining the precise location of assembly have remained elusive. Migrating T cells adopt a polarized cell shape with rear-end protrusions known as uropods. The plasma membrane-associated Gag accumulates at uropods, which participate in the formation of virological synapses, cell contact structures that play a key role in the transmission of HIV from cell to cell [34]. Ono’s lab demonstrated that Gag is associated with specific subsets of uropod-directed microdomains (UDMs) and that multimerization of Gag prior to budding was necessary for uropod localization [35]. This association of Gag with the UDMs was shown to be dependent on the polybasic region of the MA domain [36]. These data highlight the importance of the T cell uropods, and more generally the plasma membrane, as a staging area for facilitating HIV transmission and suggest a possible involvement of acidic phospholipids in this process.

Transmission of influenza virus is complicated by the fact that species-specific strains of the virus can jump hosts. For example, pigs are a well-known mixing vessel for influenza; they can be infected by strains from both birds and humans. Cross-species transmission is determined by the composition of host sialic acid receptors and the viral polymerase. As pools of divergent influenza A viruses have recently been discovered in bats, **Andrew Mehle** (University of Wisconsin) and his group are interested in understanding the potential factors necessary for bat-to-human transmission of the virus. To test this, they infected seven bat cell lines with human influenza virus or viruses expressing avian influenza polymerase. Human influenza virus was able to infect the bat cell lines via sialic acid receptors; however, viruses encoding avian polymerase replicated poorly in these bat cell lines. Serial passaging of the human influenza virus containing the avian polymerase resulted in adaption of the virus to the bat cells. Sequencing revealed a classic mutation in the avian polymerase that is the same mutation allowing avian viruses to escape restriction in humans. Passaging of the human influenza virus in the cell lines yielded a unique adaptive mutation in the polymerase. When this mutation was added back, viruses behaved like the adapted viruses. These results reveal that the influenza virus polymerase is a major driver in species-specific influenza virus adaptation.

## 7. Conclusions

Overall, the meeting was unique in that it focused on novel mechanisms of viral entry and exit across a broad range of viruses, revealing that some very distinct viruses share common strategies for spread among hosts. Attendees of this meeting gained new scientific knowledge, potential collaborations, and new methodologies, for furthering the field.

## References

[1] Doceul V., Hollinshead M., van der Linden L., Smith G.L. (2010). Repulsion of superinfecting virions: A mechanism for rapid virus spread. Science.

[2] Piccinotti S., Kirchhausen T., Whelan S.P.J. (2013). Uptake of rabies virus into epithelial cells by clathrin-mediated endocytosis depends upon actin. J. Virol..

[3] Miller E.H., Obernosterer G., Raaben M., Herbert A.S., Deffieu M.S., Krishnan A., Ndungo E., Sandesara R.G., Carette J.E., Kuehne A.I. (2012). Ebola virus entry requires the host-programmed recognition of an intracellular receptor. EMBO J..

[4] Carette J.E., Raaben M., Wong A.C., Herbert A.S., Obernosterer G., Mulherkar N., Kuehne A.I., Kranzusch P.J., Griffin A.M., Ruthel G. (2011). Ebola virus entry requires the cholesterol transporter niemann-pick c1. Nature.

[5] Evans M.J., von Hahn T., Tscherne D.M., Syder A.J., Panis M., Wölk B., Hatziioannou T., Mckeating J.A., Bieniasz P.D., Rice C.M. (2007). Claudin-1 is a hepatitis c virus co-receptor required for a late step in entry. Nature.

[6] Ploss A., Evans M.J., Gaysinskaya V.A., Panis M., You H., de Jong Y.P., Rice C.M. (2009). Human occludin is a hepatitis c virus entry factor required for infection of mouse cells. Nature.

[7] Coller K.E., Berger K.L., Heaton N.S., Cooper J.D., Yoon R., Randall G. (2009). Rna interference and single particle tracking analysis of hepatitis c virus endocytosis. PLoS Pathog..

[8] Sourisseau M., Goldman O., He W., Gori J.L., Kiem H.P., Evans V.G., Evans M.J. (2013). Hepatic cells derived from induced pluripotent stem cells of pigtail macaques support hepatitis c virus infection. Gastroenterology.

[9] Vancini R., Wang G., Ferreira D., Hernandez R., Brown D.T. (2013). Alphavirus genome delivery occurs directly at the plasma membrane in a time- and temperature-dependent process. J. Virol..

[10] Mercer J., Snijder B., Sacher R., Burkard C., Bleck C.K.E., Stahlberg H., Pelkmans L., Helenius A. (2012). Rnai screening reveals proteasome- and cullin3-dependent stages in vaccinia virus infection. Cell Rep..

[11] Kilcher S., Schmidt F.I., Schneider C., Kopf M., Helenius A., Mercer J. (2014). Sirna screen of early poxvirus genes identifies the aaa+ atpase d5 as the virus genome-uncoating factor. Cell Host Microbe.

[12] Lindenbach B.D. (2013). Virion Assembly and Release.

[13] Lindenbach B.D., Rice C.M. (2013). The ins and outs of hepatitis c virus entry and assembly. Nat. Rev. Microbiol..

[14] Domitrovic T., Movahed N., Bothner B., Matsui T., Wang Q., Doerschuk P.C., Johnson J.E. (2013). Virus assembly and maturation: Auto-regulation through allosteric molecular switches. J. Mol. Biol..

[15] Balasubramaniam M., Freed E.O. (2011). New insights into hiv assembly and trafficking. Physiology.

[16] Li F., Goila-Gaur R., Salzwedel K., Kilgore N.R., Reddick M., Matallana C., Castillo A., Zoumplis D., Martin D.E., Orenstein J.M. (2003). Pa-457: A potent hiv inhibitor that disrupts core condensation by targeting a late step in gag processing. Proc. Natl. Acad. Sci. USA.

[17] Waki K., Durell S.R., Soheilian F., Nagashima K., Butler S.L., Freed E.O. (2012). Structural and functional insights into the hiv-1 maturation inhibitor binding pocket. PLoS Pathog..

[18] Bornholdt Z.A., Noda T., Abelson D.M., Halfmann P., Wood M.R., Kawaoka Y., Saphire E.O. (2013). Structural rearrangement of ebola virus vp40 begets multiple functions in the virus life cycle. Cell.

[19] Zhang X., Guo H., Jin L., Czornyj E., Hodes A., Hui W.H., Nieh A.W., Miller J.F., Zhou Z.H., Harrison S.C. (2013). A new topology of the hk97-like fold revealed in bordetella bacteriophage by cryoem at 3.5 å resolution. eLife.

[20] Pérez-Vargas J., Krey T., Valansi C., Avinoam O., Haouz A., Jamin M., Raveh-Barak H., Podbilewicz B., Rey F.A. (2014). Structural basis of eukaryotic cell-cell fusion. Cell.

[21] Julien J.P., Cupo A., Sok D., Stanfield R.L., Lyumkis D., Deller M.C., Klasse P.J., Burton D.R., Sanders R.W., Moore J.P. (2013). Crystal structure of a soluble cleaved hiv-1 envelope trimer. Science.

[22] Lyumkis D., Julien J.P., de Val N., Cupo A., Potter C.S., Klasse P.J., Burton D.R., Sanders R.W., Moore J.P., Carragher B. (2013). Cryo-em structure of a fully glycosylated soluble cleaved hiv-1 envelope trimer. Science.

[23] Young R. (2014). Phage lysis: Three steps, three choices, one outcome. J. Microbiol..

[24] Martinez M.G., Snapp E.L., Perumal G.S., Macaluso F.P., Kielian M. (2014). Imaging the alphavirus exit pathway. J. Virol..

[25] Buckingham E.M., Carpenter J.E., Jackson W., Grose C. (2013). Autophagy and the effects of its inhibition on varicella-zoster virus glycoprotein biosynthesis and infectivity. J. Virol..

[26] Pawliczek T., Crump C.M. (2009). Herpes simplex virus type 1 production requires a functional escrt-iii complex but is independent of tsg101 and alix expression. J. Virol..

[27] Jemielity S., Wang J.J., Chan Y.K., Ahmed A.A., Li W., Monahan S., Bu X., Farzan M., Freeman G.J., Umetsu D.T. (2013). Tim-family proteins promote infection of multiple enveloped viruses through virion-associated phosphatidylserine. PLoS Pathog..

[28] Kandathil A.J., Graw F., Quinn J., Hwang H.S., Torbenson M., Perelson A.S., Ray S.C., Thomas D.L., Ribeiro R.M., Balagopal A. (2013). Use of laser capture microdissection to map hepatitis c virus-positive hepatocytes in human liver. Gastroenterology.

[29] Taylor M.P., Kobiler O., Enquist L.W. (2012). Alphaherpesvirus axon-to-cell spread involves limited virion transmission. Proc. Natl. Acad. Sci. USA.

[30] Granstedt A.E., Brunton B.W., Enquist L.W. (2013). Imaging the transport dynamics of single alphaherpesvirus particles in intact peripheral nervous system explants from infected mice. MBio.

[31] Kramer T., Enquist L. (2013). Directional spread of alphaherpesviruses in the nervous system. Viruses.

[32] Kuss S.K., Best G.T., Etheredge C.A., Pruijssers A.J., Frierson J.M., Hooper L.V., Dermody T.S., Pfeiffer J.K. (2011). Intestinal microbiota promote enteric virus replication and systemic pathogenesis. Science.

[33] Bird S.W., Maynard N.D., Covert M.W., Kirkegaard K. (2014). Nonlytic viral spread enhanced by autophagy components. Proc. Natl. Acad. Sci. USA.

[34] Jolly C. (2010). T cell polarization at the virological synapse. Viruses.

[35] Llewellyn G.N., Hogue I.B., Grover J.R., Ono A. (2010). Nucleocapsid promotes localization of hiv-1 gag to uropods that participate in virological synapses between t cells. PLoS Pathog..

[36] Llewellyn G.N., Grover J.R., Olety B., Ono A. (2013). Hiv-1 gag associates with specific uropod- directed microdomains in a manner dependent on its ma highly basic region. J. Virol..

